# Is metagenomics resolving identification of functions in microbial communities?

**DOI:** 10.1111/1751-7915.12077

**Published:** 2013-08-15

**Authors:** Ludmila Chistoserdova

**Affiliations:** Department of Chemical Engineering, University of WashingtonSeattle, WA, 98195, USA

## Abstract

We are coming up on the tenth anniversary of the broad use of the method involving whole metagenome shotgun sequencing, referred to as metagenomics. The application of this approach has definitely revolutionized microbiology and the related fields, including the realization of the importance of the human microbiome. As such, metagenomics has already provided a novel outlook on the complexity and dynamics of microbial communities that are an important part of the biosphere of the planet. Accumulation of massive amounts of sequence data also caused a surge in the development of bioinformatics tools specially designed to provide pipelines for data analysis and visualization. However, a critical outlook into the field is required to appreciate what could be and what has currently been gained from the massive sequence databases that are being generated with ever-increasing speed.

## The early days were filled with excitement. Are we there yet?

I cannot speak for everyone, but I felt that the year 2004 was a special year. This was the time when the shotgun sequencing method, by then widely used for single organism DNA sequencing, was applied to DNA from environmental samples representing the variety of organisms that form microbial communities in specific environmental niches. In short succession, two papers were published: one describing sequencing and analysis of a metagenome representing a handful of organisms forming an artificially simple community of a biofilm growing on the surface of an acid mine drainage (Tyson *et al*., 2004) and the other describing a metagenome of a much more complex community of the Sargasso Sea microbiome (Venter *et al*., 2004). While the idea itself appears to be simple these days, the demonstration that the method can actually work along with the precedents of huge investments necessary to enable such projects were very important. These early studies have been instrumental in both defining the path for the multitude of metagenomics projects that ensued, and providing the caveats for how to avoid the shortcomings of the early experiments, among them warnings about the complexity of a community in question being a defining factor in determining the depth of the sequencing effort. The functional implications of metagenomics, i.e. the importance of the connection of a specific organism/guild with a specific function as part of biogeochemical process, have been embraced from early on. A poster child of connecting a physiological feature to phylogenetic context through metagenomics remains the discovery of proteorhodopsin in bacteria (Béjà *et al*., 2000) that since resulted in a novel outlook on the potential role of proteorhodopsin-based light-driven energy flux in ocean ecosystems (DeLong and Béjà, 2010). Informed by these early studies, considerations of the complexity of connecting phylogeny to function, along with considerations of sheer cost of metagenomic sequencing, gave rise to ‘functional metagenomics’, i.e. experiments that incorporated a specific enrichment strategy or a specific technique that could target an organism/guild in question. These strategies included focusing on naturally low-complexity communities (Tyson *et al*., 2004; Hallam *et al*., 2006; Woyke *et al*., 2006), bioreactor enrichments (Strous *et al*., 2006; García Martín *et al*., 2006; Pelletier *et al*., 2008) and specific labelling strategies such as stable isotope probing (Kalyuzhnaya *et al*., 2008). In all of these cases, nearly complete genomes of novel organisms were assembled, and their physiology has been predicted in the context of a putative or known ecological function, the tasks for which availability of well-annotated genomes was a prerequisite. In our case (Kalyuzhnaya *et al*., 2008), analysis of a novel genome, the one of *Methylotenera mobilis*, has prompted significant reevaluation of the physiology of the *Methylophilaceae* that was formerly only understood based on the properties of model laboratory strains, providing a novel outlook on the ecological role of this functional guild, and directing cultivation strategies for representatives of this group that are ubiquitous but have been rarely isolated in culture (Chistoserdova, 2011). The meaningful information gleaned from these novel genomes provided optimism about the future of metagenomics and a hope for metagenomics to increasingly enable high-resolution biological knowledge in application to understanding the functionality of microbial communities and the evolutionary processes that drive their dynamics. This optimism was enhanced by the fact that individual investigators started forming consortia to more effectively address the important question of microbial ecology through metagenomics, most notable of these being the human microbiome consortium (Turnbaugh *et al*., 2007; Qin *et al*., 2010) and the soil metagenome consortium (Vogel *et al*., 2009). Meanwhile, major changes were taking place in how (meta)genomics were done. The accurate but costly Sanger sequencing technology was replaced by new-generation high-throughput technologies, dramatically decreasing the cost per base of sequence, dramatically increasing the amount of sequences generated and flooding reference databases with metagenomics-based research publications (Temperton and Giovannoni, 2012). These events, rooted in new-generation sequencing technologies, started to pose new-generation types of questions about the future of metagenomics: what is the quality of the sequences being generated, can they be processed in a meaningful way, and can we glean the functionality from the massive newly generated data so we can continue to approach some of the questions metagenomics were expected to answer in the first place, such as ‘Who is there?’, ‘What are they doing’ and most importantly, ‘Who is doing what?’ and ‘Do they do it in synergy and how?’.

## Did new-generation sequencing technologies really transform metagenomics?

From looking at the mass of publications following the switch to new-generation sequencing technology-based metagenomics, it seemed that, in a way, the field returned to time zero, being ‘caught in the headlights of new technology’ (quote from Wang *et al*., 2013), especially in terms of the connection of community function to community phylogeny. While chemistries of the new technologies are evolving to produce longer reads, with a potential to approximate the length of the Sanger sequences, and while new assembly tools produce nice results putting these together, a very common practice with these new technology-generated data has been to process unassembled reads, be it amplified 16S rRNA gene fragments or total metagenomic DNA. In the former case, only very short, ‘hypervariable’ regions are considered for comparisons, to involve as many as tens of millions of sequences (Gibbons *et al*., 2013). However, while these analyses could be done relatively quickly and in an automated fashion, the resolution of these data is very low, providing information only at the class level, thus without a strong linkage to the functional potential. For example, representatives of the class Proteobacteria are known for carrying out essentially all types of metabolism (with the exception of methanogenesis perhaps), and representatives of this class are found in, or dominate, many environments. Thus, the slice of a pie (or other graphic depiction) occupied by Proteobacteria conveys no information on what and how many metabolic functions they may be carrying out in the specific niche being addressed. This must also be true for other phyla, including the ones less represented by cultured species with known physiology. Thus, if I was told that of the 100 communities compared, all 100 (or 98) had significant proportions of Proteobacteria, what would I have learned? Time-resolved metagenomics, using the same approach, tell us that communities change over time (Caporaso *et al*., 2010; and so do the outdated low-resolution methods such as restriction fragment length polymorphism and denaturing gradient gel electrophoresis), but they do not necessarily tell us why and for what reason, as little can be gained from these analyses about the function. For example, it has been concluded from the analysis of massive data representing various mammalian metagenomes that the communities were functionally redundant (Lozupone *et al*., 2012). However, this conclusion is most likely due to the coarse-grained nature of defining a function. The same study follows to conclude that ‘Core functions of the gut microbiota include central metabolic pathways and pathways particularly important in the gut including carbohydrate and amino acid metabolism’. These are valid conclusions, but did we need massively parallel sequencing to come to them? One could predict this from just considering what is necessary for a live system to maintain itself: yes, energy and carbon metabolism, and metabolisms providing for building proteins and DNA. At the same level of ‘general function’ analysis, when compared, a human gut microbiome functional profile looks remarkably similar to the one of oxygen minimum zone marine water sample microbiome that should be (and is) a dramatically different microbiome (the two were randomly chosen by the author, and the analyses run using an automated function available through the IMG/M interface). In the terms of functional insights into respective community functions, I find this a rather disappointing result.

## Bioinformatics: what is beyond the pretty graphs?

There has been a surge in development of tools for analysing metagenomic data, and without such tools, there would not be a way of making any sense of the data. This is agreed. However, experience shows that overreliance on the tools with no access to primary sequence information could be quite dangerous (Lapidus, 2009), especially with no way (or attempt) of validating the predictions/models from automated analyses. However, these days, most scientists do not have a chance to have a look at the raw data, for the sheer volume of them, and thus they rely on the software packages, some of which are designed to process the data all the way from raw input to a variety of statistical analyses, expressed as either simple graphs or very sophisticated displays (Caporaso *et al*., 2010; Gibbons *et al*., 2013) that would make modern art museums proud were such displays made in acrylic and on large canvases. As a biologist, I still believe that the goal of bioinformatics is to help decipher biological meanings and trends as opposed to be an activity on its own. Sometimes I simply gaze at some of these displays having no idea of what they might mean. I recently reviewed a paper that was based on such ‘push-of-a-button’ analysis of microbial communities representing dramatically different soils, including pristine versus agricultural (nitrogen-impacted). This manuscript had the most beautiful graphs, but they made absolutely no sense as the analyses noted no difference between the two types of soils, whichever comparative dimension was applied. However, a large body of prior knowledge on the effect of nitrogen onto microbial communities exists (Ollivier *et al*., 2011) that disagrees with these automated analyses (and beautiful but useless graphs). As now the scene is set for comparing data from hundreds of datasets representing hundreds of time points, in a parallel fashion (Gibbons *et al*., 2013), I can only hope that some sanity checks are applied, and that we do not completely detach these sequence analyses from biology and from the main goals of metagenomics that are in understanding how microbial communities form, operate, evolve and how they drive biogeochemical cycles that keep this planet alive.

## Reality check: information from (nearly) complete genome sequences provides better clues to major microbial activities

Having expressed a fair amount of scepticism about the current state of metagenomics, I see a bright light as I follow activities of many crusaders for better metagenomics, the ones who venture beyond sorting sequences into those with and without matches to previously known genes/scaffolds and towards gaining a detailed knowledge of unknown/uncultivated. This knowledge in turn leads us towards a better understanding of how processes mediated by microbial communities work as part of our bodies or as part of biogeochemical cycles on this planet. Such ventures are indeed enabled by the new state of metagenomics when massive (gigabase-scale) data sets can be generated for each sample and analysed in a meaningful way, deciphering not only the identities of the organisms present in the sample, but connecting these, through assembling their (nearly) complete genomes, to function through reconstructing their metabolism, and ultimately through testing the predictions for their ecological function via transcriptomics, proteomics and metabolomics, and in some cases, via controlled community manipulations. I want to mention just a few exemplary studies, to prove this point. Wrighton and colleagues (2012) extracted 87 genomes from a 20 Gb data set using iterative assembly, followed by binning through self-organizing maps. Of these, 49 nearly complete genomes represent multiple lineages of uncultivated and uncharacterized bacteria belonging to five different phylum-level divisions (each of the organism types represents less than 1% of the assembled community). From the novel genomes, information is gleaned that suggests fermentative lifestyle, reliance on autotrophy via (novel, archaeal-type) RuBisCO, hydrogen production via (novel, archaeal-type) hydrogenases and sulfur reduction, metabolic strategies novel for bacteria. Representatives of one of the candidate divisions were shown to utilize a stop codon for coding tryptophan, suggesting potentially interesting evolutionary scenarios (Wrighton *et al*., 2012). An alternative approach to obtaining genome-level information on novel lineages of microbes is through sequencing genomes originating from single cells. The techniques for single-cell genomics have been dramatically improved recently, allowing for assembly of nearly complete genomes (Lasken, 2012). Swan and colleagues (2011) evaluated a total of 738 separate cells for phylogenetic markers as well as for relevant functional genes indicative of an ecological function, selecting for representatives of elusive guilds of Proteobacteria known to be ubiquitous in dark ocean but remaining uncultivated and uncharacterized. From analysis of representative genomes, they conclude on the autotrophic nature of these bacteria and identify potential sources of energy such as dissimilatory sulfur oxidation. The Deltaproteobacteria characterized in this work are the first representatives of this class containing RuBisCO, and they are also the first example of this class encoding methane metabolism functions (Swan *et al*., 2011). A combination of single-cell genome and metagenomic sequencing was applied by Dodsworth and colleagues (2013) to address the physiology of uncultivated representatives of candidate phylum OP9 and their potential role in cellulose degradation, uncovering anaerobic, fermentative, saccharolytic lifestyle (Dodsworth *et al*., 2013). Studies like the ones mentioned above truly harness the opportunities offered by the modern metagenomics and bioinformatics in order to gain new insights into the function of individual lineages as parts of complex microbial communities, while filling in gaps in genomic knowledge for major branches on the tree of life. These represent the few pieces of the extensive puzzle that nature has assembled, and metagenomics with a focus on function is one tool for solving this puzzle.
